# Detection of circulating vascular endothelial growth factor and matrix metalloproteinase-9 in non-small cell lung cancer using Luminex multiplex technology

**DOI:** 10.3892/ol.2013.1718

**Published:** 2013-11-29

**Authors:** YE ZHANG, JIAN-ZHONG WU, JUN-YING ZHANG, JING XUE, RONG MA, HAI-XIA CAO, JI-FENG FENG

**Affiliations:** 1 Department of Chemotherapy, The Affiliated Jiangsu Cancer Hospital, Nanjing Medical University, Nanjing, Jiangsu 210009, P.R. China; 2 Research Center of Clinical Oncology, The Affiliated Jiangsu Cancer Hospital, Nanjing Medical University, Nanjing, Jiangsu 210009, P.R. China; 3 Department of Oncology, Xuzhou Medical College, Xuzhou, Jiangsu 221000, P.R. China; 4 Nanjing University of Technology, Nanjing, Jiangsu 210008, P.R. China

**Keywords:** Luminex, vascular endothelial growth factor, matrix metalloproteinase-9, non-small cell lung cancer

## Abstract

It has been previously reported that vascular endothelial growth factor (VEGF) and matrix metalloproteinase (MMP)-9 are important for the occurrence and development of non-small cell lung cancer (NSCLC). The present study was designed to detect the serum levels of VEGF and MMP-9 in NSCLC, and to explore their diagnostic and prognostic values. A total of 543 cases were involved, of which 332 were NSCLC (272 cases in the pretreatment group and 60 cases in the postoperative group), 91 were patients with benign lung diseases and 120 were healthy controls. The serum levels of VEGF and MMP-9 were determined by Luminex multiplex technology. The serum levels of VEGF and MMP-9 were found to be significantly higher in the pretreatment group than those in the patients with benign lung diseases and healthy controls (VEGF, P<0.0001; MMP-9, P<0.0001). Compared with the pretreatment group, the serum levels of VEGF and MMP-9 in the postoperative group were significantly decreased (VEGF, P=0.005; MMP-9, P=0.002), and the levels of VEGF and MMP-9 in the pretreatment group of patients with stages III and IV were higher than those with stages I and II (VEGF, P<0.0001; MMP-9, P=0.021). In addition, the levels of VEGF and MMP-9 were found to closely correlate with lymph node metastasis (VEGF, P<0.0001; MMP-9, P<0.0001) in the pretreatment group, while being independent of other clinicopathological parameters (P>0.05). Furthermore, a positive correlation was observed between the serum levels of VEGF and MMP-9 (r=0.159; P=0.009). A receiver operating characteristic curve analysis showed that the diagnostic value of MMP-9 was higher than that of VEGF in the pretreatment group. The log-rank test indicated that the inoperable NSCLC patients with low levels of VEGF exhibited a significantly longer overall survival time than those with high VEGF levels (P<0.0001). Additionally, the serum levels of VEGF and lymph node metastasis were identified as independent prognostic factors of the inoperable NSCLC patients in a multivariate Cox regression analysis (P<0.05). These results indicated that VEGF and MMP-9 may be potential biomarkers for the diagnosis and prognosis of NSCLC.

## Introduction

In recent decades, lung cancer has become the leading cause of cancer-related mortality in China. In 2004, 66.7/100,000 individuals succumbed to lung cancer in China (1). Non-small cell lung cancer (NSCLC) is the principal type of lung cancer, with a 5-year survival rate of <15% (2). Therefore, the identification of specific biomarkers associated with occurrence, development and prognosis of NSCLC is essential.

In 1971, Folkman reported that new angiogenesis maintains tumor growth when the tumor is >2 mm and predicted that the presence of a tumor angiogenesis factor leads to the formation of these vessels (3). In this process, vascular endothelial growth factor (VEGF) families are the most important molecules, such as VEGF-A, -B, -C and -D and the placenta growth factor. VEGF-A, which is also called VEGF, is of great importance in angiogenesis (4).

In cancer, degradation of the extracellular matrix results in tumor cell growth, proliferation and metastasis. The matrix metalloproteinases (MMPs) represent the most critical family of endopeptidases associated with tumorigenesis. MMP-9 is a member of the MMP family and plays an evident role in the angiogenesis of tumors as well as regulating the bioavailability of VEGF (5).

The FLEXMAP 3D™ system is a multiplex platform based on suspension assay technology. The prominent advantage of this technology is that it can detect ≤500 types of analytes simultaneously in a single well. Its multiplexed function and enhanced sensitivity have been the cause of its wide use in recent years (6). In the present study, the serum levels of VEGF and MMP-9 were detected using Luminex multiplex technology, and their diagnostic and prognostic values were explored in NSCLC.

## Materials and methods

### Patients

In total*,* 332 cases of histopathologically confirmed NSCLC and 91 cases of confirmed benign lung disease were enrolled from the Affiliated Jiangsu Cancer Hospital, Nanjing Medical University (Nanjing, China) between February 2009 and November 2012. Of the NSCLC patients, 272 were classified as the pretreatment group and the remainders as the postoperative group. Initially, all the patients in the pretreatment group had been pathologically diagnosed with NSCLC and had not received any prior treatment. However, the patients in the postoperative group had received lung surgery in the previous month. The characteristics of the pretreatment group are shown in Table I; the median age of the patients was 61 years (range, 30–84 years) and all cases were staged according to the latest TNM staging issued in 2009 by the International Union Against Cancer. Of the 91 cases with benign lung diseases, 64 were pulmonary hamartomas, 17 were pulmonary inflammatory pseudotumor, six were pulmonary fibromas and four were pulmonary chondromas. The median age of the patients with benign lung dieseases was 42 years (range, 32–69 years). In addition, 120 healthy controls (without any abnormalities following a comprehensive examination) were enrolled, with a median age of 59 years (range, 35–79 years). A total of 155 inoperable NSCLC (stages IIIb and IV) patients were successfully followed up and the median survival time was 8 months (range, 1–20 months).

### Collection and preservation of blood samples

In total, 3 ml venous blood was extracted from the fasting patients and healthy controls. The blood samples were placed into the endotoxin- and pyrogen-free test tubes immediately. The whole blood specimens were then shaken three times and left to coagulate for 30 min at room temperature. Finally, the blood samples were centrifuged at 1,000 × g for 10 min, and the serum was removed and stored at −80°C prior to use. The serum of the participants was obtained following approval by the Ethics Committee of Jiangsu Cancer Hospital (Nanjing, China). Written informed consent was obtained from the patients.

### Luminex multiplex technology for VEGF and MMP-9

Luminex multiplex technology was used to conduct the present study. The FLEXMAP 3D system was supplied by Luminex Corporation (Austin, TX, USA). The serum levels of VEGF and MMP-9 were determined using human cytokine/chemokine panel (cat. no. MPXHCYTO-60K) and human cardiovascular disease panel 1 (cat. no. HCVD1-67AK) from Millipore (Billerica, MA, USA), respectively. For the main immunoassay procedure for VEGF and MMP-9, all reagents were allowed to warm to room temperature (20–25°C) prior to use. The placement of standards [0 (background), 3.2, 16, 80, 400, 2,000 and 10,000 pg/ml for VEGF; and 0 (background), 0.016, 0.08, 0.4, 2.0, 10.0, 50.0 ng/ml for MMP-9], controls 1 and 2 and samples on the Well Map Worksheet were then diagrammed in a vertical configuration. Subsequently, the filter plate was prewetted by pipetting 200 μl assay buffer into each well of the microtiter filter plate and was sealed and mixed on a plate shaker for 10 min at room temperature (20–25°C). Assay buffer was then removed by vacuum and 25 μl each standard or control was added into the appropriate wells. Assay buffer was used as the 0 pg/ml standard (background); 25 μl assay buffer was added to the sample well and 25 μl serum matrix solution was added to the background, standard and control wells. Next, 25 μl sample was added to the appropriate wells, the mixing bottle was vortexed and 25 μl beads from the kit were added to each well. Following incubation with agitation on a plate shaker overnight at 4°C, the samples were washed twice with 200 μl per well of wash buffer and 25 μl detection antibody was added to each well. After incubation with agitation on a plate shaker for 1 h at room temperature (20–25°C), 2 μl streptavidin-phycoerythrin was added to each well. The plate was then further incubated for 30 min, washed twice with 200 μl/well wash buffer and 150 μl (100 μl for MMP-9) sheath fluid was added to each well. The plate was run on the FLEXMAP 3D™system and the median fluorescence intensity results were saved and analyzed using a weighted five-parameter logistic method for calculating the concentration of samples. The assay was performed in duplicate.

### Statistical analysis

SPSS 21.0 (SPSS, Inc., Chicago, IL, USA) was used for the statistical analysis. The experimental data had a skewed distribution; therefore, non-parametric analysis was conducted. The Mann-Whitney U test was used to compare the variables between two groups, while the Kruskal-Wallis test was used to compare variables between more than two groups. The correlation between VEGF and MMP-9 was analyzed by Spearman’s rank correlation. The area under the receiver operating characteristic curve (AUROC) was calculated and the logistic regression analysis was used to evaluate the diagnostic value of the two predictors. The overall survival time was calculated as the duration between the date of the initial pathological diagnosis and mortality or the latest follow-up. The median serum levels of VEGF and MMP-9 were used as the cut-off points. The overall survival rates were estimated using the Kaplan-Meier survival curves and, simultaneously, the log-rank test was used to compare the survival distributions. Univariate and multivariate Cox regression models were used to evaluate the effect of clinicopathological parameters on patient prognosis. The median and 25–75% quartiles were used for statistical description, and P<0.05 was considered to indicate a statistically significant difference.

## Results

### Serum levels of VEGF and MMP-9 in the pretreatment group of NSCLC, patients with benign lung diseases and healthy controls

**Figure 1:**
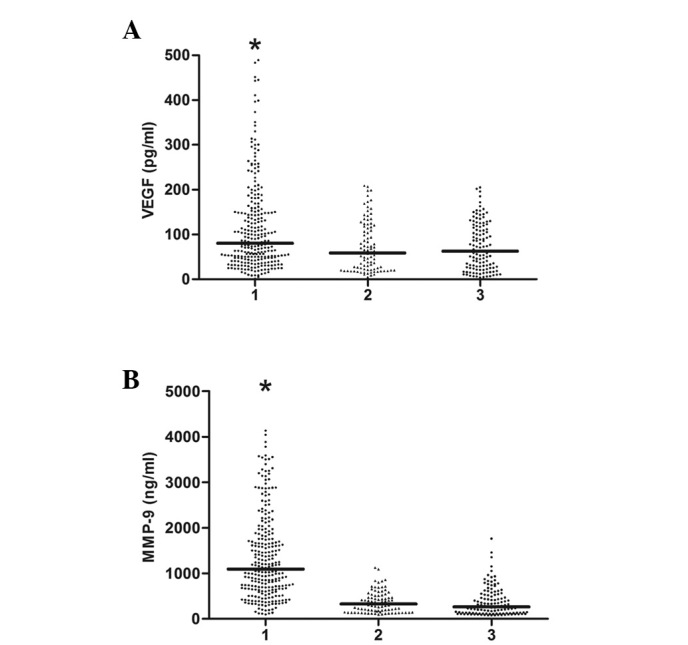
Serum levels of VEGF and MMP-9 in the pretreatment group of NSCLC, patients with benign lung diseases and healthy controls. Each dot represents an individual and the horizontal lines represent the median values. Expression of (A) VEGF and (B) MMP-9 in all groups. Groups are presented as follows: 1, pretreatment group of NSCLC; 2, patients with benign lung diseases; and 3, healthy controls. ^*^P<0.0001 vs. groups 2 and 3. VEGF, vascular endothelial growth factor; MMP-9, matrix metalloproteinase-9; NSCLC, non-small cell lung cancer.

The concentration of VEGF and MMP-9 in the serum of the pretreatment group was significantly higher than that of the patients with benign lung diseases and the healthy controls [VEGF: 81 pg/ml (range, 47–152.5 pg/ml) vs. 59 pg/ml (range, 21–118 pg/ml) and 62.5 pg/ml (range, 25.25–116.5 pg/ml), respectively (P<0.0001); and MMP-9: 1,092.5 ng/ml (range, 660.5–1,825 ng/ml) vs. 326 ng/ml (range, 169–509 ng/ml) and 259 ng/ml (range, 122.25–531 ng/ml), respectively (P<0.0001)]. However, a statistically significant difference was not observed between the patients with benign lung diseases and the healthy controls in terms of VEGF and MMP-9 serum levels (P>0.05) (Fig. 1).

### Serum levels of VEGF and MMP-9 in the pretreatment and postoperative groups of NSCLC

**Figure 2:**
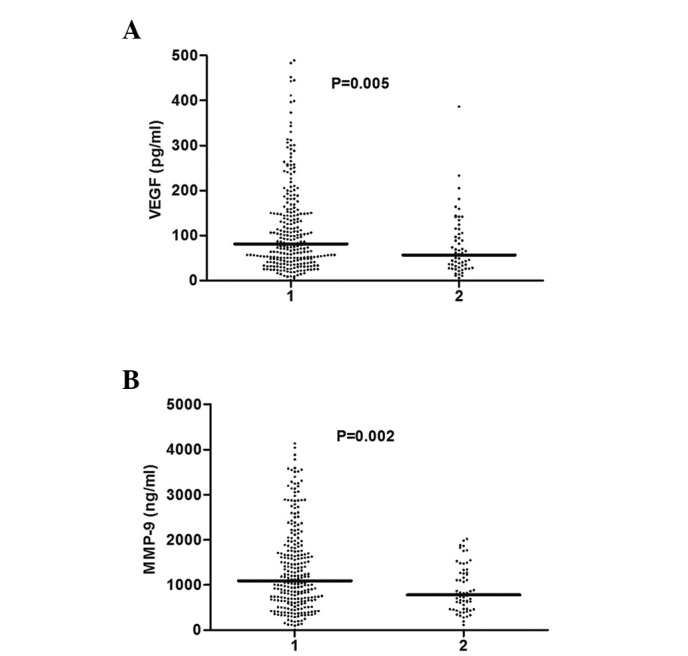
Serum levels of VEGF and MMP-9 in the pretreatment and postoperative groups of NSCLC. Each dot represents an individual and the horizontal lines represent the median values. Expression of (A) VEGF and (B) MMP-9 in the two groups. Groups are presented as follows: 1, pretreatment; and 2, postoperative. VEGF, vascular endothelial growth factor; MMP-9, matrix metalloproteinase-9; NSCLC, non-small cell lung cancer.

Compared with the pretreatment group, the serum levels of VEGF and MMP-9 were significantly decreased following the pneumonectomy [VEGF: 81 pg/ml (range, 47–152.5 pg/ml) vs. 56.5 pg/ml (range, 29.75–111.75 pg/ml), respectively (P=0.005); and MMP-9: 1,092.5 ng/ml (range, 660.5–1,825 ng/ml) vs. 778.5 ng/ml (range, 461.75–1,266.25 ng/ml), respectively (P=0.002)] (Fig. 2).

### Correlation between serum levels of VEGF and MMP-9 and clinicopathological parameters in the pretreatment group of NSCLC

**Figure 3:**
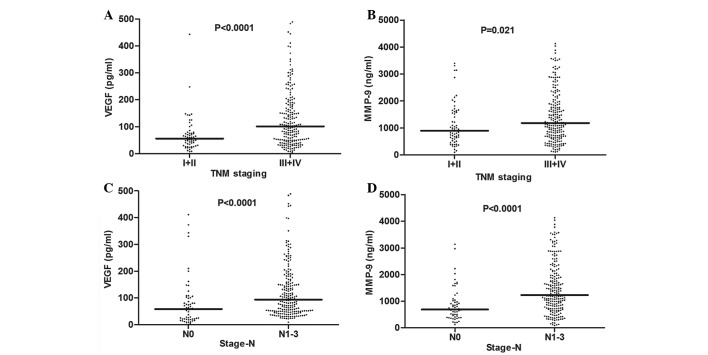
Differences between the serum levels of VEGF and MMP-9 in the pretreatment group of NSCLC according to TNM staging and lymph node metastasis (Stage-N). Each dot represents an individual and the horizontal lines represent the median values. Differences between (A) VEGF and (B) MMP-9 in the patients according to TNM staging and (C) VEGF and (D) MMP-9 in the patients according to lymph node metastasis. VEGF, vascular endothelial growth factor; MMP-9, matrix metalloproteinase-9; NSCLC, non-small cell lung cancer.

As shown in Table II, the concentration of VEGF in patients with TNM stages III and IV was higher than that in patients with stages I and II [101.5 pg/ml (range, 50–183.25 pg/ml) vs. 56.5 pg/ml (range, 34–76 pg/ml), respectively; P<0.0001] (Fig. 3A). The serum levels of VEGF were significantly higher in patients with lymph node metastasis compared with those without lymph node metastasis [94 pg/ml (range, 50–170 pg/ml) vs. 58 pg/ml (range, 20.75–101.5 pg/ml), respectively; P<0.0001] (Fig. 3C). Moreover, the levels of MMP-9 in serum was found to significantly correlate with TNM staging [1,182 ng/ml (range, 692–1,932.25 ng/ml) vs. 894 ng/ml (572–1,586.25 ng/ml), respectively; P=0.021] (Fig. 3B) and lymph node metastasis [1,299.5 ng/ml (range, 740.25–1,994.5 ng/ml), vs. 691.5 ng/ml (range, 404.75–1*,*028 ng/ml), respectively; P<0.0001] (Fig. 3D). However, no differences were identified between the serum levels of the two factors and other clinicopathological parameters (P>0.05).

### Correlation between the serum levels of VEGF and MMP-9 in NSCLC patients

**Figure 4:**
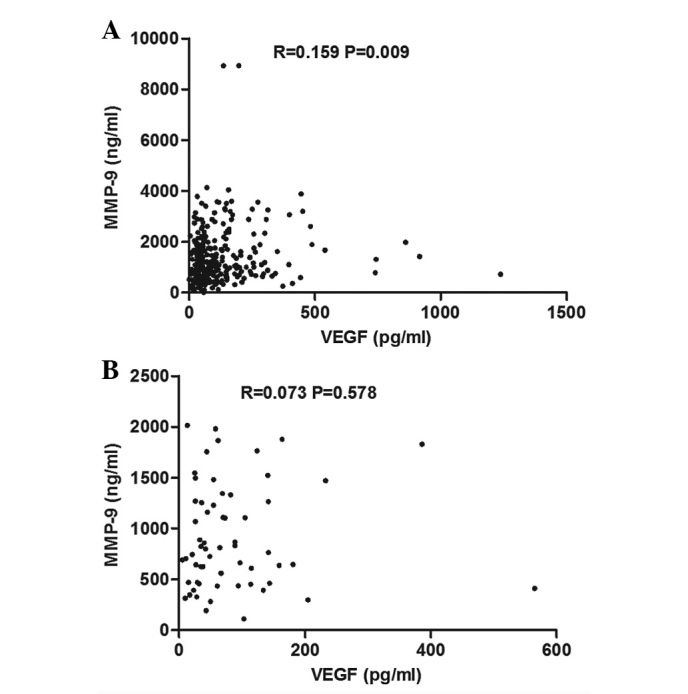
Scatter plot of the correlation between the serum levels of VEGF and MMP-9 in the (A) pretreatment and (B) postoperative NSCLC groups. VEGF, vascular endothelial growth factor; MMP-9, matrix metalloproteinase-9; NSCLC, non-small cell lung cancer.

As shown in Fig. 4, Spearman’s rank correlation was used to analyze the correlation between the serum levels of VEGF and MMP-9 in the pretreatment group of NSCLC. The results indicated that the expression of VEGF was found to significantly correlate with the expression of MMP-9 (r=0.159; P=0.009) (Fig. 4A). However, no statistically significant difference was identified between the serum levels of VEGF and MMP-9 in the postoperative group (r=0.073; P=0.578) (Fig. 4B).

### Diagnostic value of VEGF and MMP-9 in the pretreatment group of NSCLC

**Figure 5:**
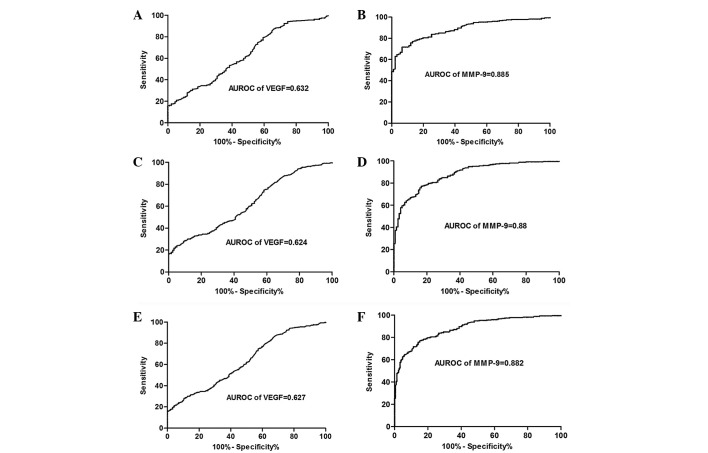
ROC curves of the serum levels of VEGF and MMP-9 for the diagnosis of NSCLC. (A and B) Pretreatment group was classified as the positive group and the patients with benign lung diseases as the negative group. (C and D) Pretreatment group was classified as the positive group and the healthy controls as the negative group. (E and F) Pretreatment group versus the benign lung disease and healthy control groups. ROC, receiver operating characteristic; AUROC, area under the receiver operating characteristic curve; VEGF, vascular endothelial growth factor; MMP-9, matrix metalloproteinase-9; NSCLC, non-small cell lung cancer.

The receiver operating characteristic curves and the logistic regression models were used to assess the diagnostic value of the serum levels of VEGF and MMP-9 in the pretreatment group of NSCLC. The AUROC of the serum levels of VEGF, differentiating the pretreatment group from the patients with benign lung diseases, was 0.632 (95% CI, 0.566–0.698) compared with 0.885 (95% CI, 0.852–0.919) for MMP-9. The AUROC of VEGF and MMP-9 was 0.624 (95% CI, 0.565–0.683) and 0.88 (95% CI, 0.845–0.914), respectively, for differentiating the pretreatment group from the healthy controls. In addition, the AUROC of VEGF for distinguishing between the pretreatment group and patients with benign lung diseases and healthy controls was 0.627 (95% CI, 0.578–0.677), while for MMP-9, the AUROC was 0.882 (95% CI, 0.853–0.911). The AUROC of MMP-9 was found to be higher than that of VEGF in the various groups. The logistic regression analysis was used to estimate the diagnostic value of the two factors and the results showed that only MMP-9 remained significant (P<0.05) (Fig. 5A–F).

### Prognostic value of VEGF and MMP-9 in the inoperable NSCLC patients

**Figure 6:**
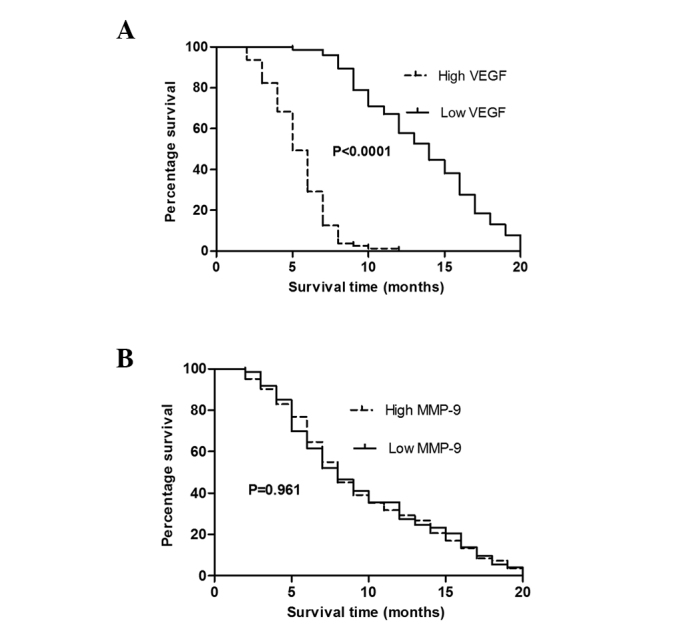
Kaplan-Meier survival curves. Survival curves of (A) VEGF and (B) MMP-9. VEGF, vascular endothelial growth factor; MMP-9, matrix metalloproteinase-9.

Kaplan-Meier survival curves and the log-rank test were used for statistical analysis in patients with inoperable NSCLC. Patients with low levels of VEGF (<101.5 pg/ml) were found to exhibit a significantly longer overall survival time than those with high levels of VEGF (>101.5 pg/ml) (P<0.0001). However, no statistically significant difference was identified in the overall survival time between the high (>1,182 ng/ml) and low levels (<1*,*182 ng/ml) of MMP-9 (P>0.05), as shown in Fig. 6. Cox regression models were used to analyze the effect of clinicopathological parameters on patient survival time, and the parameters included age, gender, levels of VEGF and MMP-9, histological type, grading, lymph node metastasis and tumor location (Table III). In the univariate Cox regression, lymph node metastasis (P=0.001) and the levels of VEGF (P<0.0001) in serum were found to closely correlate with patient survival time. The two variables were analyzed using multivariable Cox regression and the results indicated that lymph node metastasis (P=0.027) and the concentration of VEGF (P<0.0001) in serum remained significant. Therefore, the serum levels of VEGF and lymph node metastasis have been identified as independent prognostic factors in the inoperable NSCLC.

## Discussion

Neoplasm metastasis is the leading cause of mortality in patients with lung cancer. Angiogenesis and basement membrane degradation are essential in the process of the growth and invasion of tumor lesions (3,5). VEGF is important for angiogenesis, while MMP-9 is involved in basement membrane degradation. Recent studies have confirmed that VEGF stimulates the proliferation of endothelial cells by activating MMPs and, accordingly, inducing the angiogenesis of tumors (7,8). MMP-9 is critical in the invasion and metastasis of tumors and is also considered as a type of tumor angiogenic factor involved in the signaling system of VEGF-VEGF receptor (9,10). The objective of the current study was to analyze the correlation between the serum levels of VEGF and MMP-9, as well as their diagnostic and prognostic values in NSCLC.

Compared with immunohistochemical staining, the prominent advantages of measuring angiogenic factors in the blood are high availability and reduced bias (11). Luminex multiplex assays were used to detect the levels of VEGF and MMP-9 in the serum. The serum levels of VEGF and MMP-9 were found to be significantly increased in the pretreatment group of NSCLC compared with the patients with benign lung diseases and healthy controls. These results are consistent with a number of previous observations (12,13). A significant decrease in the serum levels of VEGF and MMP-9 was observed in the postoperative group compared with the pretreatment group of NSCLC patients. This result may be due to the decrease of the tumor load and suggests that the two factors are associated with the outcome of NSCLC patients.

In the present study, the serum levels of VEGF and MMP-9 were found to significantly correlate with TNM staging in the pretreatment group of NSCLC. Compared with stages I and II, the concentration of VEGF and MMP-9 in the patients with stages III and IV was significantly elevated. Currently, the expression of the two factors is detected by various methods, such as RT-PCR, immunohistochemistry and ELISA. Previous studies have shown that the expression of VEGF increases as NSCLC staging progresses (14). The expression of MMP-9 is also associated with clinicopathological stage in the serum of NSCLC (15). These hypotheses are consistent with the results of the current study. By contrast, a previous study found no correlation between the expression of the two factors and TNM staging in 91 patients with NSCLC (16).

In the present study, the serum levels of VEGF and MMP-9 in the pretreatment group of NSCLC with lymph node metastasis were significantly higher than in those without lymph node metastasis. A number of previous studies have found that the high expression of VEGF and MMP-9 closely correlate with lymph node metastasis (15,17). No significant differences were identified between the levels of the two factors and age, gender, degree of differentiation, histological type and tumor location. This result is consistent with a number of previous domestic and foreign studies (16,18–20). However, a previous study reported that the expression of VEGF and MMP-9 were found to closely correlate with the degree of differentiation, using immunohistochemical detection in 136 patients with NSCLC (21). The differential expression in the serum and tissue, as well as the use of various detection methods may account for this result. All the abovementioned studies indicate that the serum levels of VEGF and MMP-9 significantly correlate with the progression and metastasis of NSCLC.

In the current study, the correlation between VEGF and MMP-9 was analyzed and the expression of the two proteins was found to positively correlate in the pretreatment group. This result may prompt a synergistic effect of the two factors in tumor angiogenesis and lymph node metastasis in the pretreatment group of NSCLC. Previous studies have detected a significant correlation between the serum levels of VEGF and MMP-9 in patients who did not undergo surgery (7,8,22). However, no correlation between the serum levels of VEGF and MMP-9 was identified in the postoperative group of the current study. This may be due to the significant decrease in the concentration of the two indicators following tumor resection.

In the present study, it was found that the diagnostic accuracy of MMP-9 was improved compared with that of VEGF in the different groups. In the logistic regression models, only MMP-9 remained significant. The diagnostic values of VEGF and MMP-9 have remained unclear in previous studies (23,24), but certain subsidiary diagnostic values of the two indicators function to confirm NSCLC in patients who are difficult to diagnose.

Finally, the overall survival time was analyzed in the inoperable NSCLC patients of the present study. Patients with low levels of VEGF were found to exhibit a significantly longer overall survival time than those with high levels of VEGF. A large number of previous studies have reported the prognostic value of VEGF in patients with NSCLC; a large amount of data have supported the prognostic value of the expression of VEGF and have confirmed that the overexpression of VEGF is associated with poor prognosis in NSCLC (25). By contrast, a certain study was unable to identify the prognostic value of VEGF in NSCLC using quantitative immunoassay (26).

In the current study, no statistically significant difference was identified in survival time between the high and low levels of MMP-9. A previous study indicated that there was no prognostic value of the expression of MMP-9 in 90 cases of NSCLC using immunohistochemistry (27). An additional study found no correlation between the expression of MMP-9 and the overall survival time of patients with NSCLC (16). Conversely, it has been demonstrated that MMP-9 is an independent adverse prognostic factor in the tissues and serum of lung cancer (15,28). Currently, the prognostic values of VEGF and MMP-9 remain controversial.

In addition, lymph node metastasis and the pretreatment serum levels of VEGF were identified as independent prognostic variables in a multivariate analysis of the current study. Therefore, the detection of serum VEGF levels may predict the progression of tumors and aid the treatment of inoperable NSCLC patients.

Overall, the present study showed that the levels of VEGF and MMP-9 were closely correlated with the progression and metastasis of NSCLC, and that MMP-9 may be a potential diagnostic indicator of NSCLC. The pretreatment serum levels of VEGF may be an independent prognostic marker for inoperable NSCLC, and therefore may be identified as an indicator to predict the outcome of patients with inoperable NSCLC. However, there were a number of inadequacies in the current study. Firstly, the sample size was not large, therefore, future studies must be conducted in larger populations. Secondly, the follow-up was available only for inoperable patients, but not for all patients with NSCLC. All NSCLC patients must be followed up for the next five years or even decades. Finally, the evaluation of the expression of VEGF and MMP-9 was not evaluated *in situ*. An increased number of future in-depth studies are required to confirm the diagnostic and prognostic values of VEGF and MMP-9 in NSCLC.

## Figures and Tables

**Table I:** Characteristics of the pretreatment group of NSCLC.

Clinicopathological characteristics	Patients, n (%)
Gender	
Male	196 (72.1)
Female	76 (27.9)
Age, years	
>60	159 (58.5)
≤60	113 (41.5)
Tumor location	
Left lung	122 (44.9)
Right lung	148 (54.4)
Whole lung	2 (0.7)
TNM stage	
I	35 (12.9)
II	27 (9.9)
IIIa	32 (11.8)
IIIb	43 (15.8)
IV	135 (49.6)
Lymph node metastasis	
Yes	214 (78.7)
No	58 (21.3)
Grading	
1	18 (6.6)
2	128 (47.1)
3	126 (46.3)
Histological type (NSCLC)	
Squamous carcinoma	77 (28.3)
Adenocarcinoma	190 (69.9)
Adenosquamous carcinoma	4 (1.5)
Sarcoma	1 (0.3)

NSCLC, non-small cell lung cancer.

**Table II:** Correlation between serum VEGF and MMP-9 levels and clinicopathological parameters in the NSCLC pretreatment group.

	VEGF, pg/ml			MMP-9, ng/ml		
		
Variable	Median, n	Q1–Q3, n	P-value	Median, n	Q1–Q3, n	P-value
Gender						
Male	86	49.25–167.75	0.055	1159	693.5–1950.75	0.075
Female	70	34–131.25		939.5	607–1622.5	
Age, years						
>60	82	47–159	0.981	1098	660–1758	0.911
≤60	78	46.5–147		1086	665.5–1918	
Tumor location						
Left lung	79	43.5–147.25	0.203	1035.5	661.5–1699	0.150
Right lung	84	48–167.75		1160	637–1847	
Whole lung	36	22–50		2774	2036–3512	
TNM stage						
I and II	56.5	34–76	<0.0001	894	572–1586.25	0.021
III and IV	101.5	50–183.25		1182	692–1932.25	
Lymph node metastasis						
Yes	94	50–170	<0.0001	1299.5	740.25–1994.5	<0.0001
No	58	20.75–101.5		691.5	404.75–1028	
Grading						
1	154	69.25–221	0.077	998.5	587.25–3576	0.892
2	83	47.25–172		1043	636.75–2206.25	
3	73.5	43.5–131.25		1186	710.5–1603.75	
Histological type						
Squamous carcinoma	78	48–153.5	0.988	1098	725.5–2088	0.403
Adenocarcinoma	83.5	42–151.5		1092	640.25–1739.25	
Adenosquamous carcinoma	114.4	32.5–237		1308.5	736.25–1686.5	
Sarcoma^a^	74			391		

a Only one person was diagnosed with sarcoma, therefore, the value was recorded directly.

VEGF, vascular endothelial growth factor; MMP-9, matrix metalloproteinase-9; NSCLC, non-small cell lung cancer.

**Table III:** Univariate and multivariate Cox regression analysis of inoperable NSCLC patients.

	Univariate	Multivariate		
		
Variable	P-value	HR, 95% CI	Exp (B)	P-value
Age	0.299			
Gender	0.133			
VEGF	<0.0001	1.005–1.008	1.006	<0.0001
MMP-9	0.106			
Histological type	0.576			
Differentiated degree	0.232			
Lymph node metastasis	0.001	1.066–2.861	1.746	0.027
Tumor location	0.971			

HR, hazard ratio; VEGF, vascular endothelial growth factor; MMP-9, matrix metalloproteinase-9; NSCLC, non-small cell lung cancer.
